# High rates of cirrhosis and severe clinical events in patients with HBV/HDV co-infection: longitudinal analysis of a German cohort

**DOI:** 10.1186/s12876-020-1168-9

**Published:** 2020-01-30

**Authors:** Jan-Hendrik Bockmann, Marcel Grube, Vanessa Hamed, Johann von Felden, Johanna Landahl, Malte Wehmeyer, Katja Giersch, Michaela T. Hall, John M. Murray, Maura Dandri, Stefan Lüth, Ansgar W. Lohse, Marc Lütgehetmann, Julian Schulze Zur Wiesch

**Affiliations:** 1 1st Department of Internal Medicine, University Medical Hospital Hamburg-Eppendorf, Martinistr 52, 20246 Hamburg, Germany; 2 German Center for Infection Research (DZIF), Hamburg-Lübeck-Borstel site, Hamburg, Germany; 3 School of Mathematics and Statistics, UNSW Sydney, Sydney, Australia; 4 Cancer Research Division, Cancer Council NSW, Sydney, Australia; 5 Center of Internal Medicine II, University Hospital Brandenburg, Brandenburg Medical School Theodor Fontane, Brandenburg an der Havel, Germany; 6 Institute of Microbiology, Virology and Hygiene, University Medical Hospital Hamburg-Eppendorf, Hamburg, Germany

**Keywords:** HBV, HDV, HCC, Interferon, RNA, Outcome, Cirrhosis

## Abstract

**Background:**

Chronic hepatitis delta virus (HDV) infection causes severe liver disease which often leads to cirrhosis and hepatocellular carcinoma (HCC). Aim of this study was to establish the disease severity and prognostic factors for disease outcome by analysing frequencies of clinical events and their correlation with baseline virological and biochemical parameters as well as interferon and nucleos(t)ide analogue treatment choice.

**Methods:**

We studied a single-centre cohort of 49 anti-HDAg-positive patients with HBsAg persistence for at least 6 months. Virological and biochemical parameters, interferon and nucleos(t)ide analogue treatment choice as well as clinical events during follow-up were analysed by retrospective chart review (mean follow-up time 3 years, range 0.25–7.67 years).

**Results:**

Severe clinical events occurred in 11/49 hepatitis D patients, including HCC (8/49), death (8/49) or liver transplantation (2/49). HCCs only occurred secondary to liver cirrhosis and their event rates in this cohort of hepatitis D patients did not differ from a matched HBV mono-infected cohort with comparable frequency of liver cirrhosis. A stepwise multivariate logistic regression revealed low platelet count (*p* = 0. 0290) and older age (*p* = 0.0337) correlating most strongly with overall clinical events, while serum HDV RNA positivity at baseline did not correlate with any clinical outcome. Interferon-free but not nucleos(t)ide analogue-free patient care correlated with the occurrence of HCC at logistic regression, although only 3/18 interferon-treated patients demonstrated repeatedly negative HDV PCR results post therapy.

**Conclusions:**

Our data indicate that progressive liver disease at baseline plays a major role as predictive factor for overall clinical outcome of hepatitis D patients. In particular, HCC risk may not be underestimated in hepatitis D virus RNA negative hepatitis D patients with advanced liver fibrosis.

## Background

Approximately 62–72 million people worldwide are chronically coinfected with hepatitis delta virus (HDV) and hepatitis B virus (HBV) [1]. Compared to chronic hepatitis B and C virus mono-infection, HDV coinfection is associated with higher rates of liver cirrhosis, hepatocellular carcinoma (HCC) [2–6] and liver related mortality [7]. Chronic HDV infection is still challenging. While nucleos(t) ide analogues (NAs) do not exhibit any direct antiviral efficacy against HDV, pegylated interferon-alpha (IFN) is the only approved HDV treatment option [8]. Previous randomized trials could demonstrate HDV RNA negativity in 20–30% of treated patients 24 weeks after the end of IFN treatment [9, 10]. However, due to high relapse rates during follow-up a “sustained virological response” is only achieved in the minority of patients [9]. Despite the low rates of treatment response, recent studies suggest that IFN therapy has a favourable effect on disease progression [3, 11–13]. Overall, data on factors affecting outcome of chronic HDV patients in the real-life setting are still limited.

The aim of this study was to longitudinally analyse the individual disease course including clinical and virological parameters of chronic HDV patients at our university centre focusing on factors associated with long-term clinical outcome.

## Methods

In the current study we screened 651 HBV infected patients attending the University Medical Center Hamburg-Eppendorf between January 2008 and June 2016 for anti-HDV positivity. Inclusion criteria were positive HBsAg status as well as positive anti-HDV immunoglobulin results for at least 6 months. Patients who had been liver transplanted or showed history of HCC before baseline were excluded from the study. The methods for diagnosing liver cirrhosis, virological measurements and statistical analysis can be found in the Additional file 1. The study was approved by the local ethics board (Ethik-Kommission der Ärztekammer Hamburg, WF-035/17).

## Results

In total, 49 hepatitis D patients were included for subsequent analysis, the mean follow-up time of these 49 patients was 3 years (range 0.25–7.67 years). 34/49 patients showed evidence of HDV replication and serum HDV levels > 100 U/ml at baseline, while the remaining 15 patients showed negative serum HDV RNA at baseline and repeatedly during follow-up. Eighteen HDV RNA positive patients started IFN therapy within the observation period. 25/49 (51%) hepatitis D patients were on anti-HBV therapy with NUCs at baseline and none of the 49 patients had previously received anti-HDV IFN therapy before baseline. The baseline characteristics of the hepatitis D cohort are summarized in Additional file 2: Table S1. Fibroscan or liver biopsy was performed in all 49 HDV patients at baseline. Overall, more than a third (18/49, 36.73%) of patients were cirrhotic at baseline, which was substantially higher than in the HBV-mono-infected cohort, where 50/602 hepatitis B patients (8.31%) showed evidence of liver cirrhosis at the beginning of the follow-up period. Three patients of the current hepatitis D cohort had a concomitant HIV infection, additional information of these three patients are shown in Additional file 3: Table S2. None of the 49 hepatitis D patients showed evidence of active HCV co-infection.

Severe clinical events (HCC, death, liver transplantation) occurred at a relatively high frequency of 22.45% (11/49 patients) during follow-up. In contrast, severe clinical events only occurred in 14/602 (2.33%) of the HBV mono-infected patients. The frequency of liver cirrhosis as a main risk factor of development of HCC was higher in the group of HDV infected patients (36.73%) compared to HBV mono-infected patients (8.31%). However, when the group of hepatitis D patients was matched by age and frequency of cirrhosis to a similar HBV mono-infected group using propensity score matching [14], the occurrence of total events did not significantly differ between HBV-HDV-coinfected (11/49, 22.45%) and HBV mono-infected (7/49, 14.29%) patients.

**Fig. 1:**
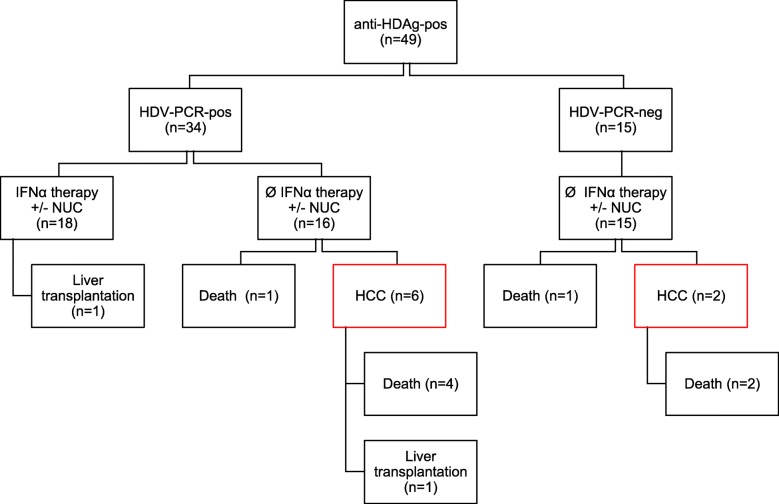
Patient outcome of Hepatitis D patients. Severe clinical events included death, HCC development and liver transplantation

Interestingly, 10 of the 11 hepatitis D patients with severe events where in the group that did not receive IFN therapy. When this was normalized to the length of the  observation period, the annual HCC incidence was 0.055/ hepatitis D patient. All HCCs developed secondary to liver cirrhosis. Eight out of 18 (44.44%) cirrhotic patients developed HCCs- 4 of them at the stage of compensated cirrhosis and 4 at the stage of decompensated cirrhosis. None of the IFN-treated patients developed HCC or died during follow-up, while 2 HDV RNA negative patients without IFN treatment and 6 patients with detectable HDV RNA at baseline but without IFN treatment developed HCCs during follow-up (Fig. 1).

Univariate logistic regression analysis of the entire cohort revealed that non-initiation of an IFN therapy correlated with the occurrence of total events, HCC and mortality (*p* = 0.0379, *p* = 0.0198 and *p* = 0.0198), but not with occurrence of liver transplantation. Interestingly, NA treatment positively correlated with total events, HCC and mortality, reflecting that cirrhotic patients were treated with NAs more frequently than non-cirrhotic patients. Furthermore, baseline levels of age, low platelet counts, INR, MELD score and bilirubin were significantly associated with total clinical events and HCC rates. With the exception of age, the indicated parameters also correlated with mortality, whereas none of the parameters correlated with liver transplantation. Interestingly, baseline HBV and HDV virological (HBV DNA, HDV RNA, HDV RNA positivity, HBsAg and HDV genotype) parameters did not correlate with the number of total or HCC events (Table 1), except for HBeAg positivity correlating with the number of total events (*p* = 0. 0422) as well as mortality (*p* = 0. 0065). A stepwise multivariate logistic regression revealed low platelet count (*p* = 0. 0290) and older age (*p* = 0.0337) as the main correlates of overall clinical events.

**Table 1:** Correlation between baseline parameters and occurrence of HCCs, mortality, liver transplantation and total clinical events during follow-up analysed by univariate logistic regression

	HCC	Mortality	Liver transplantation	Total events
Cirrhosis	0.0014	0.0150	1.0000	0.0046
Male sex	0.4106	0.4106	1.0000	0.4663
NUC treatment	0.0042	0.0497	0.4902	0.0049
Interferon treatment	0.0198	0.0198	1.0000	0.0379
HDV PCR positive	1.0000	0.6869	1.0000	1.0000
HDV genotype	0.4104	0.4104	0.9891	0.7423
HDV-RNA (IU/ml)	0.8004	0.1446	0.4018	0.3493
HBV-DNA (IU/ml)	0.7417	0.9507	0.6855	0.8138
HBeAg-pos	0.1001	0.0065	1.0000	0.0422
HBsAg (IU/ml)	0.9633	0.7851	0.9642	0.8285
Albumin (g/L)	0.1222	0.1291	0.9412	0.2454
INR	0.0057	0.0181	0.0878	0.0037
Platelet count (10^9^/L)	0.0063	0.0259	0.1436	0.0036
Bilirubin (mg/dl)	0.0203	0.0019	0.8724	0.0027
MELD score	0.0062	0.0092	0.1153	0.0026
ALT (U/L)	0.5156	0.6844	0.6294	0.5339
AST (U/L)	0.6483	0.3476	0.9362	0.3322
yGT (U/L)	0.4852	0.4343	0.1767	0.2102
Age	0.0880	0.0633	0.7643	0.0395

**Fig. 2:**
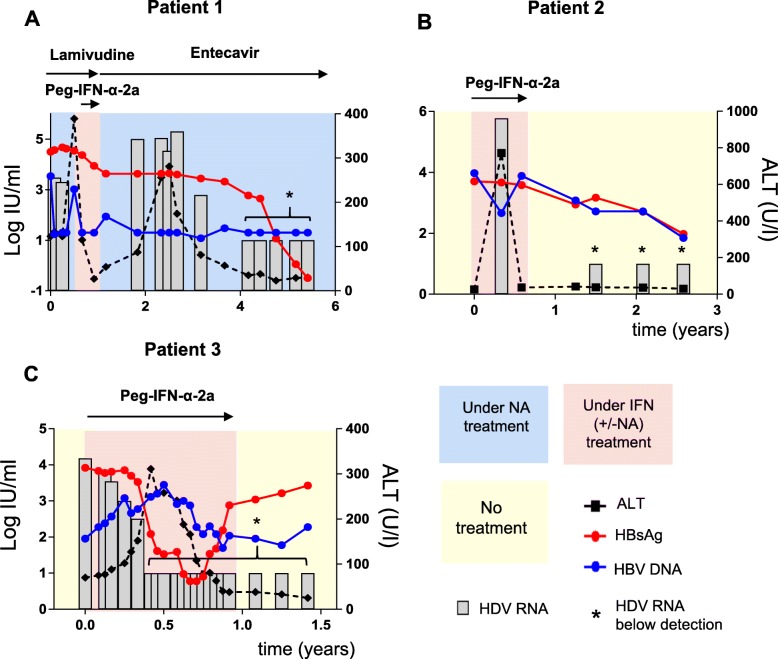
Virological and biochemical courses of 3/18 patients responding to IFN therapy by serum HDV RNA, HBV DNA, HBsAg and ALT levels

Eighteen of the 49 hepatitis D patients were treated with IFN in order to achieve either HBsAg seroconversion or HDV RNA negativity according to the EASL guidelines 2009–2017 [15]. None of the patients had been treated with IFN before the observation period as indicated in Fig. 2, Additional file 4: Figure S1 and Additional file 5: Figure S2. The median IFN treatment duration within the 18 treated patients was 48 weeks. Seven patients (7/18, 39%) had to discontinue the minimum treatment duration of 48 weeks for various reasons (e.g. patient’s request, thrombocytopenia, provider’s individual decision). In line with previous studies that have reported high relapse rates of serum HDV RNA after IFN therapies, there were considerable fluctuations of serum HDV RNA within single courses of IFN treatment (Fig. 2, Additional file 4: Figure S1 and Additional file 5: Figure S2) and reoccurrence of temporarily negative HDV viremia after IFN treatment in 5/18 patients.

Therefore, in our analysis we avoided to use the term “sustained virological response” as defined end point of a HDV IFN treatment. Instead, in order to adequately describe the HDV treatment response to IFN therapy, repeatedly negative HDV viremia results (≥ three PCR measurements at an interval of at least 12 weeks) after the end of IFN therapy were used. Virological data for this analysis were available for 17/18 (94%) IFN-treated patients. Three out of 18 patients (ITT: 17%, PP: 18%) repeatedly showed negative HDV-PCR results in the long-term follow-up after IFN therapy. Figure 2 shows the detailed clinical courses of these three patients. HDV PCR levels of patient 1 became negative at later time points after IFN therapy (4 years) and subsequent initiation of therapy with entecavir. Patient 2 and 3 had not been treated with NAs and displayed negative HDV PCR results early after cessation of IFN therapy (patient 2: 1.5 years after initiation of IFN therapy) or early during IFN therapy (patient 3: 0.4 years). Interestingly, these three patients with reproducible negative HDV PCR results also showed a decrease in serum HBsAg during follow-up, while none of the other IFN-treated patients showed any decrease of HBsAg (Additional file 4: Figure S1 and Additional file 5: Figure S2). The early decline of HDV RNA in patient 2 and 3 was closely linked to the decline of HBsAg. On the contrary, a modest HBsAg decline that could be seen in patient 1 preceded the HDV RNA decline by 2 years. HBsAg seroconversion could not be observed in any of the patients so far. However, decline of HDV RNA was closely associated with normalization of liver transaminases in patients 1–3.

## Discussion

The current study describes the clinical outcome of hepatitis D patients in a single- centre cohort and during long-term follow-up. The study shows a high rate of hepatocellular carcinomas (16%, 8/49, mean observation period 3 years) in hepatitis D patients which is in the upper range of previously reported HCC rates that varied from 3 to 15% [3, 5, 6, 11, 12]. Interestingly, all HCCs in hepatitis D patients occurred secondary to liver cirrhosis. Although the HCC rate was much higher compared to HBV mono-infected patients at our centre, this difference (HCC rate or total events) disappeared, when matched cohorts with comparable frequency of liver cirrhosis were compared. In the multivariate logistic regression analysis only low platelets counts and higher age at baseline remained significant predictive factors for total events (HCC, death, liver transplantation). Interestingly, neither the level of hepatitis D viremia nor the HDV RNA status (positive or negative) did significantly correlate with outcome. Therefore, our data would indicate that the high rate of HCCs in hepatitis D patients is mainly caused by rapid progression to liver cirrhosis per se and not by viral activity at baseline. Thus, our data indicate that HCC risk in HDV RNA negative patients with progressive liver disease may be underestimated.

Over 50% of the patients received NUC therapy at baseline or during follow-up to supress HBV replication. However, absence of NUC therapy did not correlate with occurrence of clinical events, indicating that control of HBV replication does not improve the outcome of hepatitis D patients.

Interestingly, IFN-free patient care correlated with the occurrence of HCCs. Nevertheless, according to the retrospective design, IFN-untreated patients showed higher frequencies of decompensated liver cirrhosis and 3 cases of HIV coinfection, therefore these patients had a priori a higher risk of mortality and HCC development. However, it is important to note that -even after exclusion of the 3 HIV patients-, an absence of IFN therapy still correlated with the occurrence of HCC in the univariate logistic regression. These data are in line with the results from a study by Farci et al., which suggested lower HCC rates in HDV infected patients treated with IFN compared to untreated patients [14]. Wranke et al. observed beneficial effects of IFN therapy on the overall outcome of HDV patients [11]. Conversely, other studies challenged the hypothesis of IFN as a HCC protective agent [7, 13].

In our cohort, 37% of the patients were treated with IFN. The long-term HDV RNA negativity rate achieved by IFN treatment was 17% which is lower than those rates reported in previous studies (20–30% 24 weeks after end of IFN therapy) [10]. However, the long-term response rate remained fairly comparable with the long-term HDV RNA negativity rate of around 17% analysed by Heidrich et al. [9] and Boglione et al. [16].

Nevertheless, the antiviral effects with regards to HBV infection appeared to be worse than previously described [11, 16], since none of the IFN-treated patients reached HBsAg seroconversion. These disappointing antiviral responses of IFN-treated HDV infected patients on the one hand, and the high HCC risk and HCC-related mortality due to progressive liver disease -including HDV PCR negative patients- on the other hand, emphasise the urgent need for novel therapeutic strategies for HDV infection [17].

## Conclusions

The present study confirms the high morbidity of HDV/HBV-infected patients in a German cohort with high rates of cirrhosis and high rates of clinical events. Low platelet counts and high age can serve as predictors for poor overall clinical outcome. Our results caution to carefully screen anti-HDV- and HBsAg-positive patients for liver-related events regardless of hepatitis D viremia.

## Supplementary information

**Additional file 1.** Supplementary materials.

**Additional file 2: Table S1.** Baseline characteristics of hepatitis D patients (*n* = 49), total hepatitis B mono-infected patients (*n* = 602) and hepatitis B patients matched to hepatitis D patients for cirrhosis and age by propensitiy score (*n* = 49).

**Additional file 3: Table S2.** Baseline characteristics of HBV-HDV-HIV coinfected patients.

**Additional file 4: Figure S1.** Selected cases of patients not responding to IFN therapy indicated by courses for serum HDV RNA, HBV DNA, HBsAg and ALT levels.

**Additional file 5: Figure S2.** Selected cases of patients not responding to IFN therapy indicated by courses for serum HDV RNA, HBV DNA, HBsAg and ALT levels.

## Data Availability

The datasets used and/or analysed during the current study are available from the corresponding author upon reasonable request.

## Abbreviations

ALT: Alanine aminotransferase
AST: Aspartate aminotransferase
HBeAg: Hepatitis B early antigen
HBsAg: Hepatitis B surface antigen
HBV: Hepatitis B virus
HCC: Hepatocellular carcinoma
HDV: Hepatitis delta virus
IFN: Pegylated interferon-alpha-2a
INR: International normalized ratio
NUC: Nucleoside or nucleotide analogue
