# Supplementary material for: High rates of cirrhosis and severe clinical events in patients with HBV/HDV co-infection: longitudinal analysis of a German cohort
Source: BMC Gastroenterol. 2020 Jan 30;20:24. doi: 10.1186/s12876-020-1168-9 (PMC6993357; doi:10.1186/s12876-020-1168-9)
Supplement: Supplementary file 1 — Additional file 1. Supplementary materials. [file 12876_2020_1168_MOESM1_ESM.docx]

Additional file 1

**Methods**

**Diagnosis of liver fibrosis**

Diagnosis of liver cirrhosis was based on liver histology (F4 according to the Desmet score), or by transient elastography (≥13 kilo pascal) using a FibroScan (EchoSens, Paris, France). Briefly, median liver stiffness in kilopascal was calculated by at least 10 valid measurements in a 4-6cm target area of the right liver lobe. Only measurements with an interquartile range/median ratio of less than 30% were considered as valid ([1](#_ENREF_1)). Hepatocellular carcinoma was diagnosed according to the respective national guideline either by biopsy or cross sectional imaging with computed tomography or magnetic resonance imaging scan ([2](#_ENREF_2), [3](#_ENREF_3)).

**Virological parameters**

Viral RNA and DNA was extracted from EDTA-plasma using the QiAsymphony SP (Qiagen, Hilden Germany) automated extraction system. HBV DNA levels were quantified by HBV RealStar Assay (Altona Diagnostics, Hamburg, Germany) using PEI reference material (#3620/05) for normalization to IU/ml. LLOD and LLOQ of the HBV DNA PCR were 3.8 IU/ml and 12 IU/ml, respectively. HDV RNA levels were quantified by a modified one-step PCR assay as described by Ferns et al ([4](#_ENREF_4)) and normalized to the first HDV WHO standard (PEI # 7657/12); the lower detection limit of the HDV PCR was 100 IU/ml. HDV RNA serum levels before 2012 were assessed by a PCR protocol by LeGal et al ([5](#_ENREF_5)). HDV genotyping was performed as previously described ([6](#_ENREF_6)) using qScript XLT One - Step RT (Quanta Biosciences, USA). Phylogenetic analysis of HDV sequences was performed using eight prototype HDV sequences retrieved from the NCBI GenBank as published ([7](#_ENREF_7)). Quantitative HBsAg was measured with the Abbott Architect Analyzer 2000sr system (Abbott, Germany) and qualitative HBeAg levels were determined with the Siemens Centauer XP system. Qualitative anti-HDV levels were analysed using a competitive enzyme immunoassay (Diasorin, Saluggia, Italy).

**Statistical analysis**

Statistical analysis was performed with SPSS and Matlab R2017a, and figures were created with the GraphPad Prism 5 software. The association between parameters at baseline and disease-related clinical events was analysed by univariate logistic regression. A multivariate logistic regression with a step-wise fit was performed for non-categorical baseline parameters to assess the association between clinical outcomes and combinations of baseline parameters. A value of p <0.05 was considered statistically significant.

1. Sandrin L, Fourquet B, Hasquenoph JM, Yon S, Fournier C, Mal F, et al. Transient elastography: a new noninvasive method for assessment of hepatic fibrosis. Ultrasound Med Biol. 2003;29(12):1705-13.

2. European Association For The Study Of The L, European Organisation For R, Treatment Of C. EASL-EORTC clinical practice guidelines: management of hepatocellular carcinoma. Journal of hepatology. 2012;56(4):908-43.

3. Heimbach JK, Kulik LM, Finn R, Sirlin CB, Abecassis M, Roberts LR, et al. Aasld guidelines for the treatment of hepatocellular carcinoma. Hepatology. 2017.

4. Ferns RB, Nastouli E, Garson JA. Quantitation of hepatitis delta virus using a single-step internally controlled real-time RT-qPCR and a full-length genomic RNA calibration standard. Journal of virological methods. 2012;179(1):189-94.

5. Le Gal F, Gordien E, Affolabi D, Hanslik T, Alloui C, Deny P, et al. Quantification of hepatitis delta virus RNA in serum by consensus real-time PCR indicates different patterns of virological response to interferon therapy in chronically infected patients. Journal of clinical microbiology. 2005;43(5):2363-9.

6. Ivaniushina V, Radjef N, Alexeeva M, Gault E, Semenov S, Salhi M, et al. Hepatitis delta virus genotypes I and II cocirculate in an endemic area of Yakutia, Russia. The Journal of general virology. 2001;82(Pt 11):2709-18.

7. Nguyen HM, Sy BT, Trung NT, Hoan NX, Wedemeyer H, Velavan TP, et al. Prevalence and genotype distribution of hepatitis delta virus among chronic hepatitis B carriers in Central Vietnam. PloS one. 2017;12(4):e0175304.
