# Supplementary material for: High rates of cirrhosis and severe clinical events in patients with HBV/HDV co-infection: longitudinal analysis of a German cohort
Source: BMC Gastroenterol. 2020 Jan 30;20:24. doi: 10.1186/s12876-020-1168-9 (PMC6993357; doi:10.1186/s12876-020-1168-9)
Supplement: Supplementary file 2 — Additional file 2: Table S1. Baseline characteristics of hepatitis D patients (n = 49), total hepatitis B mono-infected patients (n = 602) and hepatitis B patients matched to hepatitis D patients for cirrhosis and age by propensitiy score (n = 49). [file 12876_2020_1168_MOESM2_ESM.docx]

Additional file 2: **Table S1:** Baseline characteristics of hepatitis D patients (n=49), total hepatitis B mono-infected patients (n=602) and hepatitis B patients matched to hepatitis D patients for cirrhosis and age by propensitiy score (n=49).

|  | **Hepatitis D (n=49)** | **Hepatitis B (n=602)** | **Hepatitis B matched (n=49)** |
| --- | --- | --- | --- |
| **Male sex** | 33 (67%) | 351 (58%) | 48 (98%) |
| **Median age, years (IQR)** | 44 (36.75-52) | 42 (33.25-52) | 51 (38-63) |
| **Cirrhosis** | 18 (37%) | 50 (8%) | 18 (37%) |
| **HIV positive** | 3 (6%) | 0 (0%) | 0 (0%) |
| **NUC treatment** | 28 (57%) | n=225 (37%) | 33 (67%) |
| **Median HDV-RNA, IU/ml (IQR)** | 1.0x 10^5^ (8.5 x 10^3^-5.6x 10^5^) | - | - |
| **Median HBV-DNA, IU/ml (IQR)** | 1.5x 10^2^ (1.0 x 10^2^-8.2x 10^3^) | 1.7x 10^3^ (1.2 x 10^1^-5.2x 10^4^) | 5.2x 10^2^ (1.2 x 10^1^-2.6x 10^5^) |
| **HBeAg-pos** | 8 (16%) | 56 (9%) | 6 (12%) |
| **HBsAg, IU/ml (IQR)** | 7.6x 10^3^ (2.0 x 10^3^-1.3x 10^4^) | 5.05x 10^3^ (5.0 x 10^2^-1.4x 10^4^) | 6,2x 10^3^ (5.0 x 10^2^-1.7x 10^4^) |
| **HDV genotype** | 18x GT 1  2x GT 5  2x GT8 | - | - |
| **Median Albumin, g/L (IQR)** | 38.5 (34-42.75) | 43 (40-46) | 45 (40-48) |
| **Median INR, (IQR)** | 1.11 (1.04-1.22) | 1,02 (0.97-1.08) | 1,08 (1.05-1.22) |
| **Median Platelet count,10^9^/L (IQR)** | 162 (99-197) | 217 (177-260) | 192 (87-225) |
| **Median Creatinine, mg/dl (IQR)** | 0.8 (0.7-0.98) | 0.8 (0.7-0.96) | 0.89 (0.8-1.05) |
| **Median ALT, U/L (IQR)** | 69 (44-101) | 31 (22-53) | 47 (30-67) |
