# Supplementary material for: High rates of cirrhosis and severe clinical events in patients with HBV/HDV co-infection: longitudinal analysis of a German cohort
Source: BMC Gastroenterol. 2020 Jan 30;20:24. doi: 10.1186/s12876-020-1168-9 (PMC6993357; doi:10.1186/s12876-020-1168-9)
Supplement: Supplementary file 3 — Additional file 3: Table S2. Baseline characteristics of HBV-HDV-HIV coinfected patients. [file 12876_2020_1168_MOESM3_ESM.docx]

Additional file 3: **Table S2:** Baseline characteristics of HBV-HDV-HIV coinfected patients

|  | **Patient 1** | **Patient 2** | **Patient 3** |
| --- | --- | --- | --- |
| **CD4 cell count (cells/µl)** | 201 | 573 | 471 |
| **HIV PCR (cop/ml)** | 76 | undetectable | undetectable |
| **HBV PCR (IU/ml)** | 190 | undetectable | undetectable |
| **HDV PCR baseline (IU/ml)** | 1.7x 10^8^ | 1.0x 10^2^ | 6.0x 10^4^ |
| **HIV subtype** | 1 | 2 | 1 |
| **ART since baseline** | yes | yes | yes |
