# Supplementary material for: High rates of cirrhosis and severe clinical events in patients with HBV/HDV co-infection: longitudinal analysis of a German cohort
Source: BMC Gastroenterol. 2020 Jan 30;20:24. doi: 10.1186/s12876-020-1168-9 (PMC6993357; doi:10.1186/s12876-020-1168-9)
Supplement: Supplementary file 4 — Additional file 4: Figure S1. Selected cases of patients not responding to IFN therapy indicated by courses for serum HDV RNA, HBV DNA, HBsAg and ALT levels. [file 12876_2020_1168_MOESM4_ESM.pptx]

## Slide 1
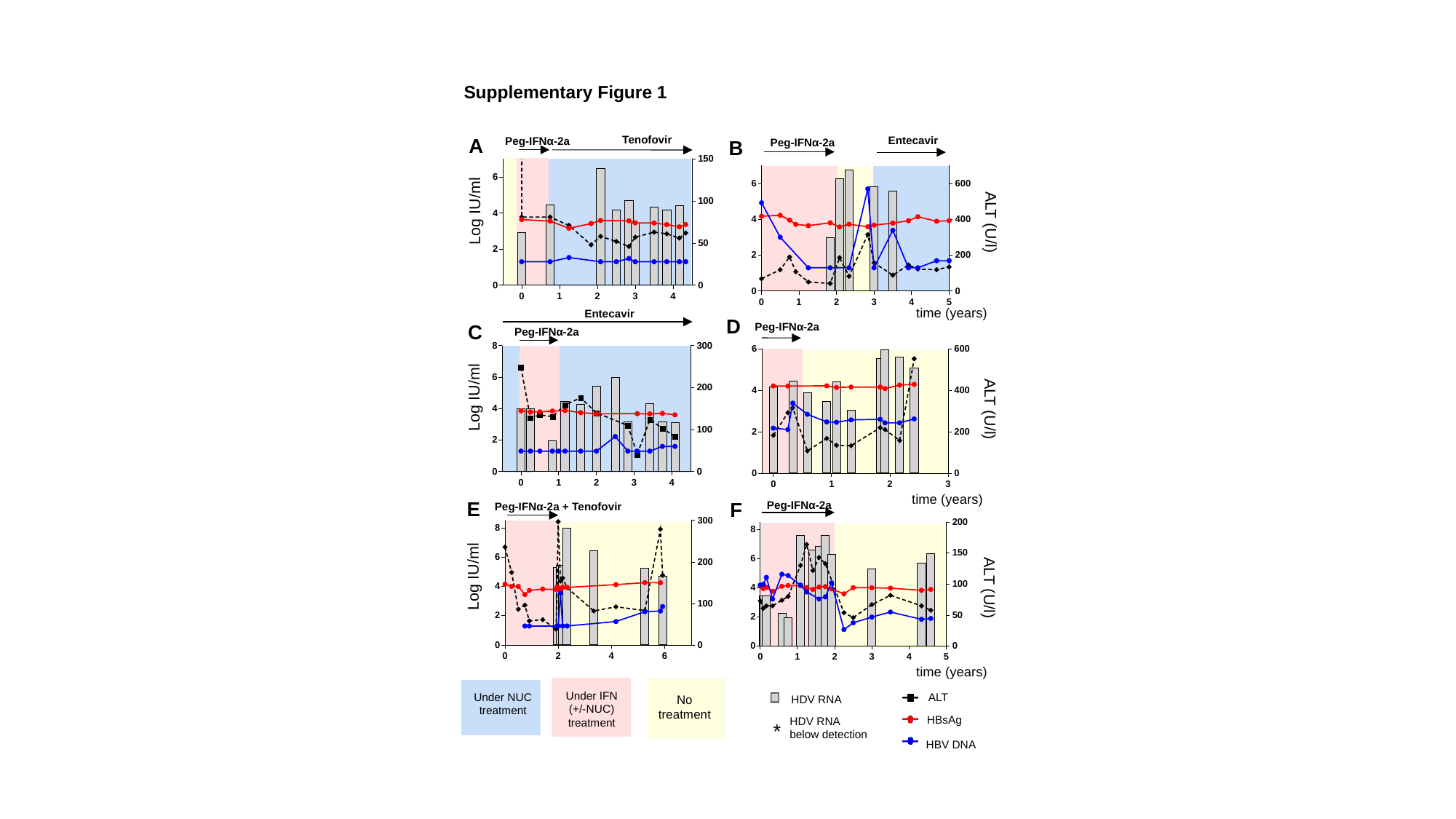

Supplementary Figure 1
Tenofovir
A
Entecavir
Peg-IFNα-2a
Peg-IFNα-2a
B
Log IU/ml
ALT (U/l)
time (years)
Entecavir
D
Peg-IFNα-2a
C
Peg-IFNα-2a
Log IU/ml
ALT (U/l)
time (years)
E
F
Peg-IFNα-2a
Peg-IFNα-2a + Tenofovir
Log IU/ml
ALT (U/l)
time (years)
Under IFN
(+/-NUC) treatment
Under NUC treatment
ALT
No treatment
HDV RNA
HBsAg
 HDV RNA
 below detection
*
HBV DNA
