# Supplementary material for: High rates of cirrhosis and severe clinical events in patients with HBV/HDV co-infection: longitudinal analysis of a German cohort
Source: BMC Gastroenterol. 2020 Jan 30;20:24. doi: 10.1186/s12876-020-1168-9 (PMC6993357; doi:10.1186/s12876-020-1168-9)
Supplement: Supplementary file 5 — Additional file 5: Figure S2. Selected cases of patients not responding to IFN therapy indicated by courses for serum HDV RNA, HBV DNA, HBsAg and ALT levels. [file 12876_2020_1168_MOESM5_ESM.pptx]

## Slide 1
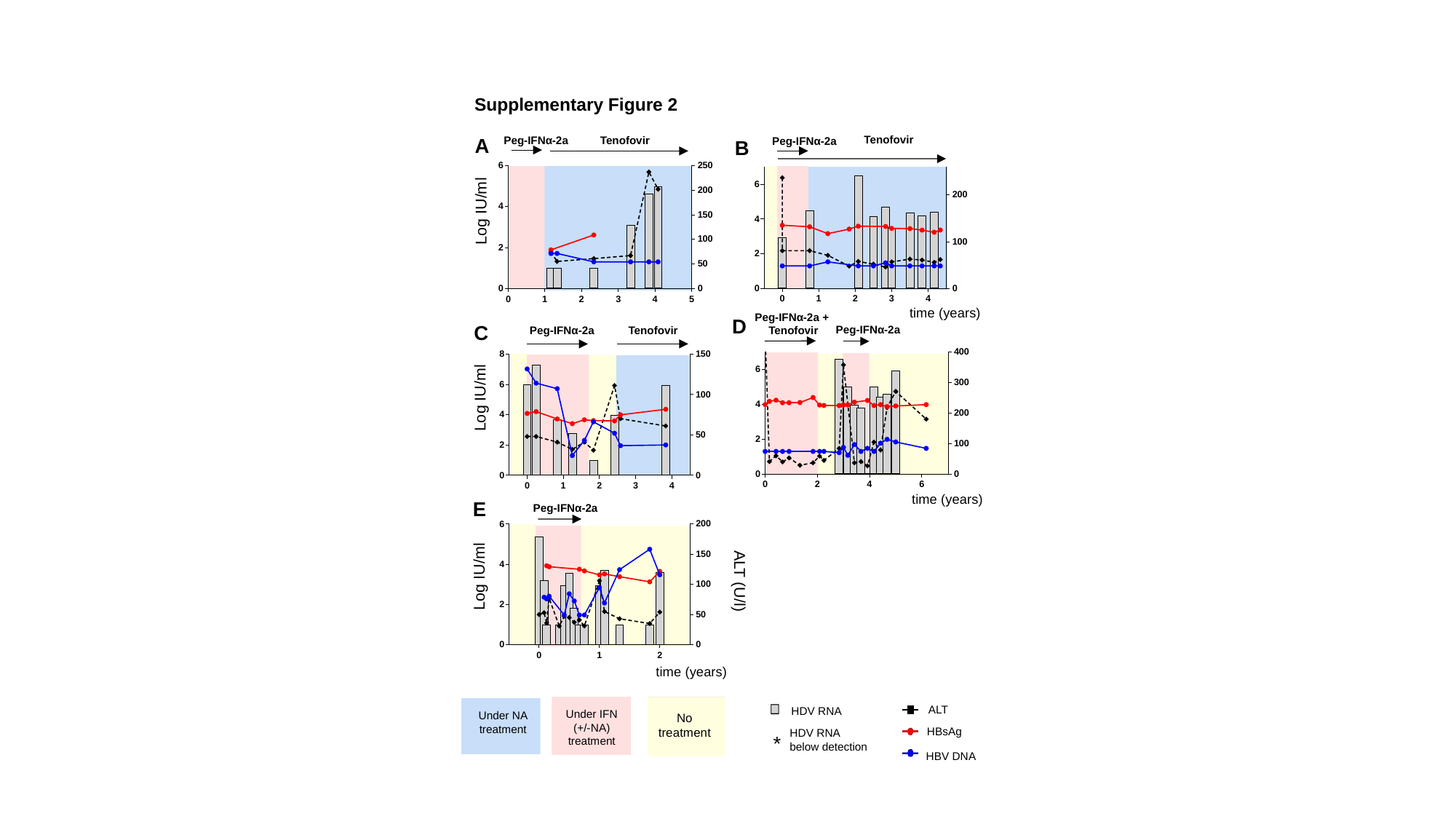

Supplementary Figure 2
Tenofovir
A
Tenofovir
Peg-IFNα-2a
Peg-IFNα-2a
B
Log IU/ml
time (years)
Peg-IFNα-2a +
Tenofovir
D
C
Peg-IFNα-2a
Tenofovir
Peg-IFNα-2a
Log IU/ml
time (years)
E
Peg-IFNα-2a
Log IU/ml
ALT (U/l)
time (years)
ALT
HDV RNA
Under IFN
(+/-NA) treatment
Under NA treatment
No treatment
HBsAg
 HDV RNA
 below detection
*
HBV DNA
